# Perfusion Pressure Deficits as Early Indicators of Abdominal Compartment Syndrome After Neonatal Congenital Diaphragmatic Hernia Repair: A Retrospective Cohort Study

**DOI:** 10.3390/life16091496

**Published:** 2026-09-07

**Authors:** Raluca-Alina Gogolan, Catalin-Gabriel Cirstoveanu, Ana-Mihaela Bizubac, Mariana-Carmen Heriseanu, Carmina Nedelcu, Adrian-Iustin Georgevici, Nicolae Sebastian Ionescu

**Affiliations:** 1 Doctoral School, “Carol Davila” University of Medicine and Pharmacy, 050474 Bucharest, Romania; raluca-alina.mocanu@drd.umfcd.ro (R.-A.G.); carmen.iliescu@drd.umfcd.ro (M.-C.H.); carmina.georgescu@drd.umfcd.ro (C.N.); 2 Department of Pediatric Surgery, “Maria Sklodowska Curie” Emergency Clinical Hospital for Children, 041451 Bucharest, Romania; sebastian.ionescu@umfcd.ro; 3 Department of Neonatal Intensive Care, “Carol Davila” University of Medicine and Pharmacy, 050474 Bucharest, Romania; mihaela.bizubac@umfcd.ro; 4 Neonatal Intensive Care Unit, “Maria Sklodowska Curie” Emergency Clinical Hospital for Children, 041451 Bucharest, Romania; 5 Department of Anesthesiology, St. Josef Hospital of Ruhr-University of Bochum, 44791 Bochum, Germany; adrian-iustin.georgevici@rub.de; 6 Department of Pediatric Surgery and Orthopedics, “Carol Davila” University of Medicine and Pharmacy, 050474 Bucharest, Romania; 7 Romanian Academy of Medical Sciences, 030171 Bucharest, Romania; 8 Romanian Academy of Scientists, 010071 Bucharest, Romania

**Keywords:** congenital diaphragmatic hernia, intra-abdominal pressure, abdominal perfusion pressure, abdominal compartment syndrome

## Abstract

(1) Background: Abdominal compartment syndrome (ACS) after congenital diaphragmatic hernia (CDH) repair is an infrequent but potentially life-threatening consequence of viscero-abdominal disproportion. Because static intra-abdominal pressure (IAP) thresholds may not capture hypoperfusion, this study assessed whether early postoperative deficits in mean arterial pressure (MAP) and abdominal perfusion pressure (APP) could serve as early indicators of ACS. (2) Methods: We performed a retrospective cohort analysis of neonates with CDH admitted between January 2022 and June 2025. APP was calculated as MAP minus IAP. Two deficit features were defined: the maximum decrease in APP below 45 mmHg and MAP below 55 mmHg within the 72 h after surgical repair. A penalized logistic regression model with leave-one-out cross-validation assessed discriminative ability. (3) Results: Among 28 patients, two developed ACS. Median minimum MAP and APP were lower in the ACS group (35.7 mmHg and 27.7 mmHg, respectively) than in the non-ACS group (48 mmHg and 40.3 mmHg, respectively). The penalized model achieved an exploratory ROC-AUC of 0.942 (95% CI 0.808–1). Deficit features were computed before clinical decompensation. (4) Conclusions: Early MAP and APP deficits may provide physiologic warning signal for ACS after CDH, warranting prospective multicenter validation.

## 1. Introduction

Although seldom observed, abdominal compartment syndrome (ACS) after congenital diaphragmatic hernia (CDH) repair can result in significant morbidity. Delayed abdominal fascial closure in CDH patients has been reported in the literature; however, it has not been described as a consequence of increased intra-abdominal pressure (IAP) [1,2]. This suggests that the potential impact of elevated IAP, despite its clinical significance, remains under-recognized in the surgical literature. Greater awareness of this overlooked factor is crucial, as increased IAP may significantly influence postoperative outcomes in CDH patients.

Increased IAP and ACS following gastroschisis repair due to viscero-abdominal disproportion have been well described [3], leading to a recommendation to measure IAP in patients with congenital abdominal wall defects [4,5]. Extrapolating the impact of viscero-abdominal disproportion on IAP, similar monitoring should be considered following congenital diaphragmatic hernia repair.

Commonly, ACS is described as an increased IAP above 20 mmHg associated with a new organ failure [6], but these thresholds were derived from adult data. In 2013, the World Society of the Abdominal Compartment Syndrome (WSACS) adjusted the consensus definitions for the pediatric population, stipulating that ACS is defined as IAP > 10 mmHg correlated with new or exacerbated organ dysfunction [7].

IAP can be measured directly, using intraperitoneal piezoresistive probes, or indirectly, measuring the pressure in the inferior vena cava, stomach, bladder, uterus or rectum [8,9]. Studies demonstrate that intravesical pressure is an accurate reflection of IAP [8,9,10,11,12], and current WSACS guidelines recommend this technique of IAP measurement [7]. Trans-vesical IAP measurement is particularly challenging in neonates because the saline volumes instilled in the bladder and the degree of head elevation in bed may alter the IAP measurement [13,14,15].

Pediatric reports, including prospective studies and systematic reviews, suggest that clinically significant hypoperfusion can occur despite IAP values falling within the normal range, especially when the mean arterial pressure (MAP) is borderline [16,17]. Consequently, abdominal perfusion pressure (APP = MAP − IAP) could provide a more reliable indicator of organ perfusion, preceding ACS, as supported by studies assessing the renal and gut perfusion and cardiovascular effects of increased IAP [18,19]. While Horoz et al. demonstrated the APP is a useful tool of predicting mortality in a pediatric intensive care population, CDH-specific data are lacking [20]. Furthermore, it is well known that pulmonary hypoplasia and pulmonary hypertension occur in congenital diaphragmatic hernia requiring significant ventilator support [21]. Considering that elevated ventilatory pressures have been demonstrated to decrease cardiac output through increased intrathoracic pressure, they may also result in low MAP [22,23].

Observations synthesized from pediatric review publications emphasize the need for identifying predictors of ACS development and for evidence-based treatment protocols in order to improve outcomes [16,24,25].

We hypothesized that postoperative perfusion deficits, defined as decreases in MAP and APP below physiologic safety thresholds, could identify neonates at risk of ACS within the first 72 h after CDH repair.

## 2. Materials and Methods

### 2.1. Study Design

We performed a retrospective cohort study of neonates with CDH admitted from January 2022 to June 2025 at a tertiary-level academic pediatric emergency hospital. This study is reported in accordance with the Strengthening the Reporting of Observational Studies in Epidemiology (STROBE) guidelines.

Inclusion criteria: Eligibility required undergoing surgical correction of CDH, continuous hemodynamic monitoring and intraoperative and postoperative measurement of IAP for a minimum of three postoperative days. No formal sample size calculation was performed; all eligible patients during the study period were included.

Exclusion criteria: We excluded patients who died prior to surgery and those with severely compromised clinical status who died within hours after surgery.

In our institution, written informed consent is obtained from the parents of all the subjects upon admission. Our informed consent form contains paragraphs addressing data use in research studies and participation in clinical teaching, given the university hospital setting. Furthermore, the study was approved by the hospital’s Ethics Committee.

### 2.2. Data Collection

Data were extracted from patients’ medical records, operative records and bedside charts.

We developed a database that included demographic variables (sex, gestational age, birth weight, mode of delivery, Apgar score, prenatal diagnosis status), surgical variables (type of congenital diaphragmatic defect, liver herniation in the thoracic cavity, surgical approach, repair technique, time from birth until surgical procedure) and postoperative parameters (trans-vesical IAP, oxygen saturation, types of ventilatory support and their associated parameters—peak inspiratory pressure for conventional mechanical ventilation and mean airway pressure for High-Frequency Oscillatory Ventilation, heart rate, arterial pressure, diuresis, presence of intestinal transit, presence of edema, the number of days until enteral feeding and length of hospital stay.

Personally identifiable information was not included in our database.

### 2.3. Measurement of IAP

IAP measurement followed the WSACS recommendations for children [7]. We positioned the patient supine without elevation of the head of the bed. We connected a three-way stopcock to the Foley catheter. One port of the stopcock was attached to the urine collecting bag, and the other port was connected to a pressure transducer attached to the multiparameter patient monitor. After “zero calibration” of the transducer, we instilled 1 mL/kg (minimum 3 mL) of sterile room-temperature NaCl 0.9% solution in the bladder and recorded the pressure, expressed in mmHg, displayed on the multiparameter patient monitor.

### 2.4. Outcome Definition

The primary outcome was clinician-adjudicated postoperative ACS within seven days of repair. ACS was adjudicated according to the WSACS consensus pediatric definitions (sustained IAP elevation >10 mmHg with new renal, respiratory, or cardiovascular dysfunction) [4]. Organ dysfunction was assessed based on clinical parameters including decreased urine output, increased ventilatory requirements, and increased vasoactive support requirement. IAP was measured via the urinary catheter as previously described in Section 2.3. The assessment integrated the bedside documentation, charted MAP and IAP values, and creatinine and lactate level trends. The event timing was aligned to the first sustained elevation fulfilling WSACS criteria to anchor risk calculations. Adjudication was performed by the clinical team based on prospectively documented bedside data. The seven-day outcome window was chosen to capture delayed ACS presentations while maintaining clinical relevance; the three-day predictor window captures the early postoperative period when perfusion deficits are most likely to manifest.

The secondary endpoints included evaluating the association of IAP, MAP and APP with ACS and decompression requirement.

### 2.5. Predictor Definition

Postoperative day (POD) 0 was defined as the calendar day of surgery. The analytic window spanned the first three postoperative days (POD 0–2). MAP readings originated from invasive arterial catheters with documentation governed by unit protocols.

For each infant, we recorded the lowest daily-mean MAP and APP during the first three postoperative days. If the minimum APP fell below the 45 mmHg target, the shortfall (45 minus the observed minimum) was recorded as the APP deficit; infants whose APP never dropped below 45 mmHg received a deficit of zero. The MAP deficit was defined analogously using a 55 mmHg target. These thresholds reflect conservative neonatal perfusion targets derived from neonatal critical care practice and pediatric perfusion studies. For the gestational age range in the present cohort (34–40 weeks), normative MAP values place 55 mmHg between the 50th and 75th percentiles [26,27]. This threshold may nonetheless require adjustment for preterm infants at the lower end of the gestational age range. The APP threshold of 45 mmHg represents MAP 55 mmHg minus the WSACS pediatric IAH threshold of 10 mmHg [7] and is a constructed threshold that has not been empirically validated in neonates.

For patients who developed ACS and required emergent decompression, only MAP and APP values recorded prior to the clinical diagnosis or surgical intervention were used for predictor computation, ensuring that the deficit features preceded the outcome rather than reflecting decompensation during established ACS.

Required predictor variables (deficit features) were complete for all infants; MAP and APP minimum values were unavailable for one non-ACS infant, due to incomplete data, but this did not affect deficit computation. No imputation or multivariable adjustment beyond the prespecified deficits was undertaken.

### 2.6. Statistical Analysis

The primary analysis used a penalized (ridge) logistic regression, a method demonstrated to perform well for risk estimation with few events [28], with the two deficit features as predictors. Because APP is mathematically derived from MAP (APP = MAP − IAP), the two deficit predictors are inherently collinear; penalized (ridge) regression was chosen in part because it natively accommodates multicollinearity without numerical instability, distributing the shared signal across correlated predictors rather than requiring one to be dropped. The model performance was assessed using leave-one-out cross-validation (LOOCV) to account for the small sample. The penalization parameter (lambda) was selected via an inner cross-validation loop nested within the LOOCV procedure. Features were standardized using training-fold means and standard deviations, and the final coefficients were back-transformed to raw measurement units for reporting. Discrimination was summarized with the area under the receiver operating characteristic curve (ROC-AUC) with percentile bootstrap 95% confidence intervals (2000 replicates). Wilcoxon rank-sum tests compared deficit distributions between ACS and non-ACS groups. With 2 ACS events and two predictors, the events-per-parameter ratio was 1, which falls below the recommended minimum of five to ten events per parameter for penalized models [29,30] and well below the minimum sample size derived from modern prediction model frameworks [31]. We therefore emphasize penalization and LOOCV to mitigate overfitting, report confidence intervals to communicate uncertainty, and frame these results as exploratory. As sensitivity analyses, we also fit a Bayesian generalized linear model with additional physiologic covariates and performed decision-curve analysis [32,33], measurement-noise sensitivity analysis, and variable-importance network analysis (Supplementary Materials). Model fitting was performed using a Python 3.14.0 pipeline (scikit-learn for ridge regression, NumPy/SciPy for Bayesian inference); all reporting, tables, and figures were generated in R 4.3.1 using the targets workflow engine, with flextable, ggplot2, and ggrepel as key packages.

## 3. Results

**Figure 1:**
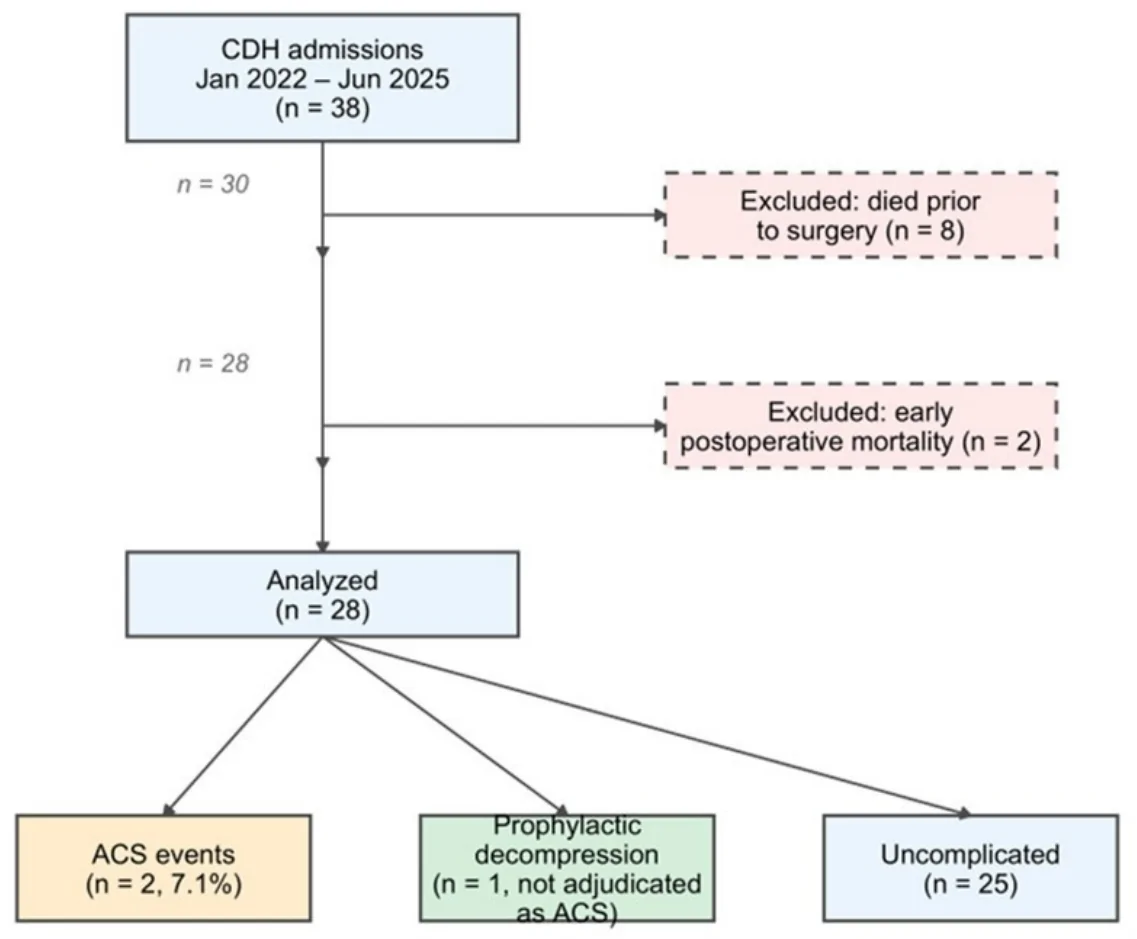
Patient flow diagram. CDH, congenital diaphragmatic hernia; ACS, abdominal compartment syndrome.

Throughout the period of study, there were 38 patients admitted for CDH, but we excluded from the study eight patients who died prior to surgery and two patients with severely compromised general status who died in the early postoperative period, within 24 h after surgery (at 1 and 10 h postoperatively, respectively). All 28 eligible neonates were analyzed with no post hoc exclusions. Two cases of ACS events were observed, corresponding to an observed frequency of 0.071. Figure 1 details the patient selection.

Table 1 summarizes the baseline characteristics for the overall cohort and by ACS status. Among the 28 patients included in the study, 17 were male, and 11 were female. The median gestational age and the median birth weight were 38 weeks and 3080 g, respectively. Five-minute Apgar scores were depressed in ACS infants (4.5 vs. 7), reflecting the well-documented association between neonatal depression and postoperative instability [34,35]. Overall, 89.3% of patients were diagnosed prenatally, and cesarean delivery accounted for 78.6% of births.

The time from birth to surgical intervention ranged from 10 to 216 h, with a median of 30.5 h (IQR 22.8–48.0) and a mean of 43.2 h. Of the 28 patients included in the study, five had right-sided CDH, and 23 had left sided CDH. The surgical approach was determined according to the surgeon’s preference and technical expertise. Twenty-four patients underwent open abdominal CDH repair, and four patients underwent thoracoscopic repair. Regarding the size of the posterolateral diaphragmatic defect, two patients had type A defects, 11 type B, 13 type C and two type D, according to the CDH Study Group classification [36]. Liver herniation into the thoracic cavity was observed intraoperatively in 11 patients. Notably, liver herniation was observed in both patients who developed ACS. Prosthetic diaphragmatic reconstruction was performed in nine infants (32.1%), concentrated among ACS events (50% vs. 30.8%), aligning with older series describing increased abdominal tension after patch closure of the diaphragm [25,34,35]. Prophylactic decompression was documented in one infant (3.6%), reflecting pre-emptive patch closure of the abdominal wall due to observed intraoperative tension.

Both ACS infants had markedly lower MAP and APP minima than the remainder of the cohort. The median minimum MAP was 48 mmHg in the non-ACS group versus 35.7 mmHg among ACS cases (individual values: 33 mmHg and 38.3); similarly, the median minimum APP was 40.3 mmHg versus 27.7 mmHg (individual values: 22 mmHg and 33.3 mmHg). The corresponding deficits were substantially higher in ACS cases (MAP deficit 19.3 in ACS group vs. 6.3 mmHg in non-ACS group; APP deficit 17.3 vs. 4.7 mmHg).

**Figure 2:**
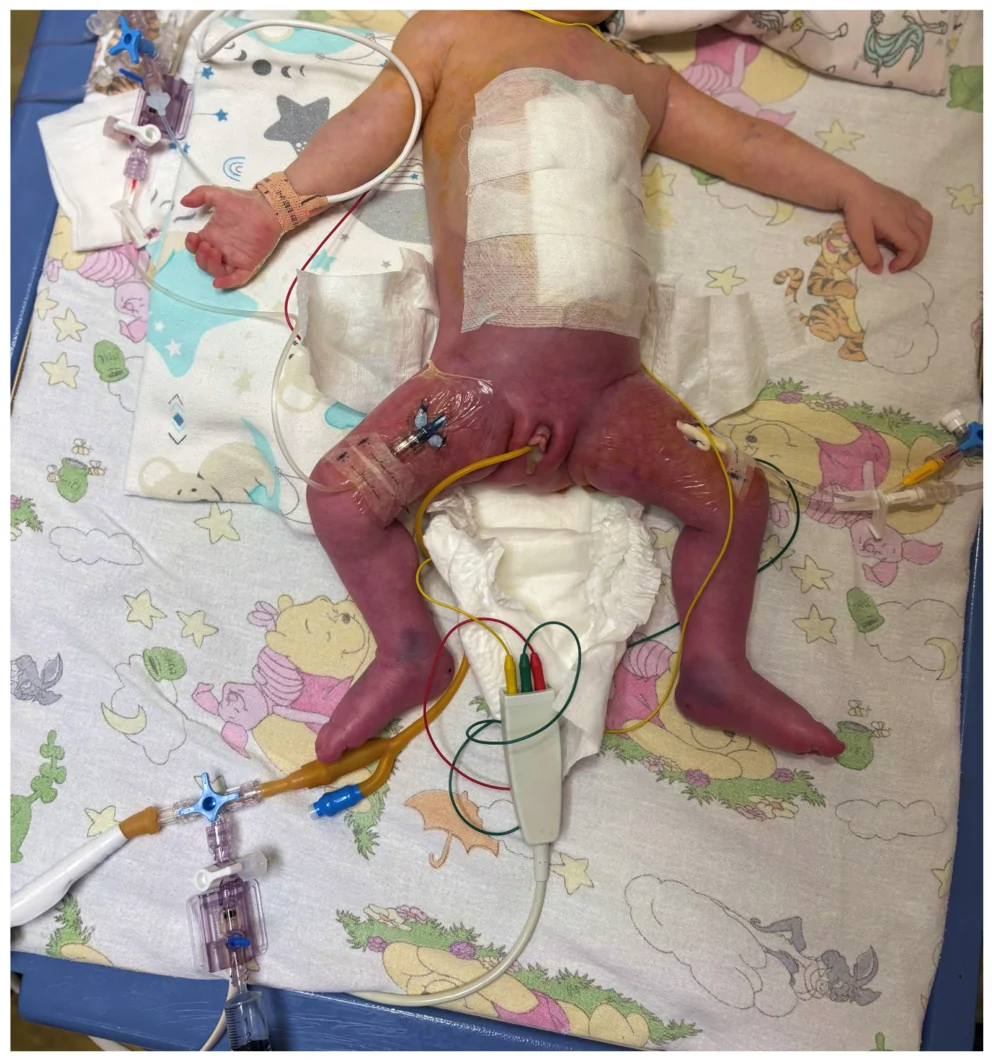
Pre-decompression clinical appearance of the lower extremities, suggestive of impaired venous return and peripheral perfusion in the setting of abdominal compartment syndrome, with resolution following decompression.

In one case (patient 23), clinical ACS was diagnosed based on progressive organ dysfunction (renal and respiratory compromise), despite an early postoperative recorded IAP of 8 mmHg, consistent with reports that ACS can occur at IAP values below formal thresholds when the MAP is critically low [16,17]. Patient 37 underwent prophylactic patch placement due to observed intraoperative abdominal tension before pressure recordings and was not adjudicated as an ACS event; this case represents a potential outcome misclassification if the intervention pre-empted ACS that would otherwise have occurred. In patient 46, ACS was clinically diagnosed at 6 h postoperatively (Figure 2) and subsequently confirmed by intravesical pressure measurement, which showed a value of 20 mmHg (Figure S4—Supplementary Materials) prior to decompression.

The penalized logistic model achieved an ROC-AUC 0.942 (95% CI 0.808–1), indicating apparent discrimination, though this metric is unreliable with only two events. For each additional mmHg that APP fell below 45, the odds of ACS increased (Table 2). A Bayesian companion model (Supplementary Table S1) and decision-curve analysis (Supplementary Figure S1) are presented in the Supplementary Materials.

**Figure 3:**
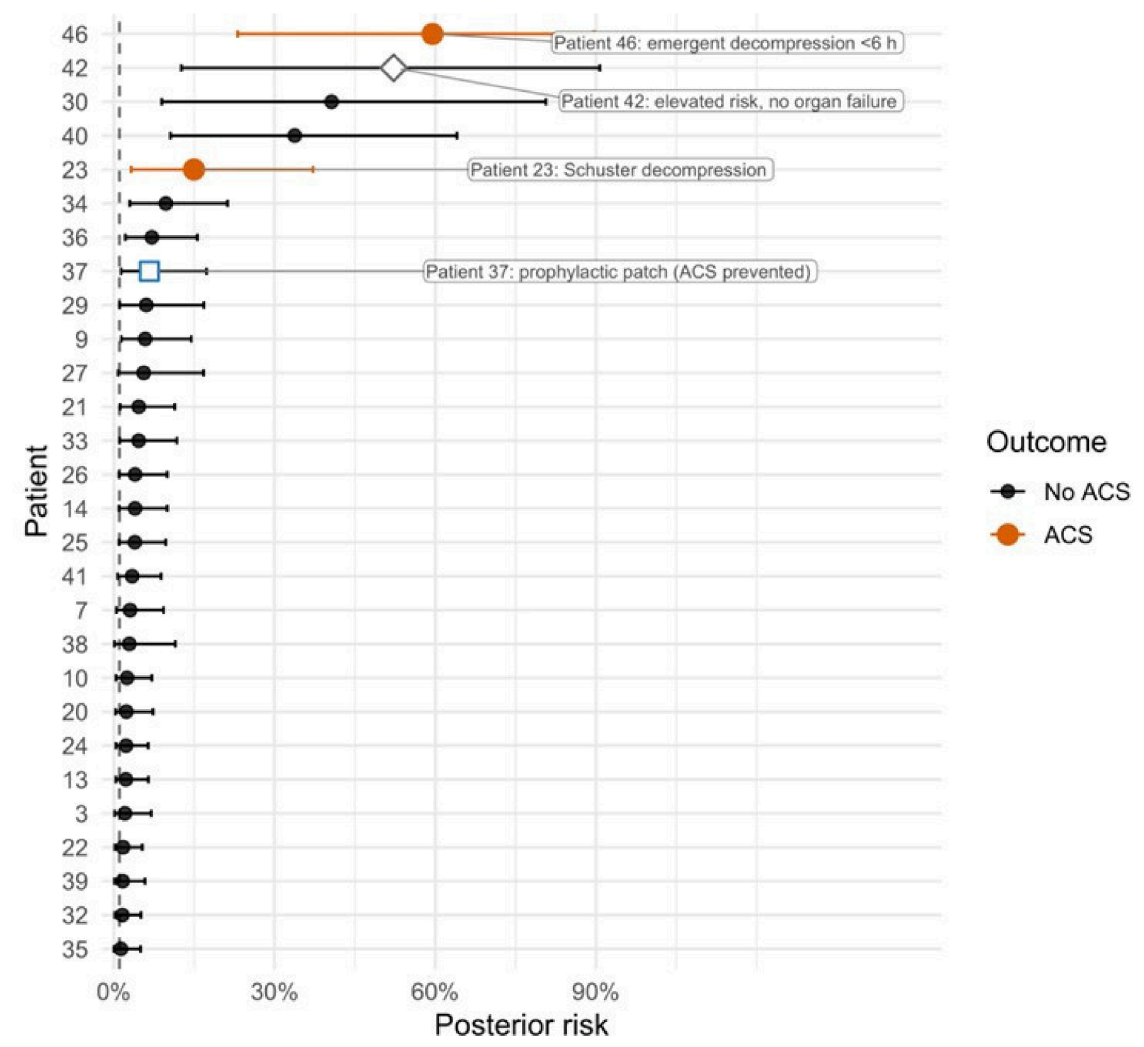
Posterior ACS risk per patient with 90% credible intervals. Adjudicated ACS cases (patients 23 and 46), the prophylactically decompressed patient 37, and the elevated-risk non-event patient 42 are annotated. The dashed line marks the alert threshold.

Posterior risks concentrated below 5% for non-events, while the two adjudicated ACS cases sat above the clinical alert band with credible intervals separated from the decision threshold (Figure 3). Patient 46 ranked highest with a posterior mean of 0.595 (90% CrI 0.231–0.896); this infant underwent emergent Silobag decompression less than six hours post-repair. Patient 23, who underwent Schuster decompression postoperative day 1, showed a similarly elevated risk (0.149). The low MAP in patient 23 may partly reflect primary hemodynamic compromise (Apgar 4) rather than compartment physiology alone. Patient 37, whose tense closure prompted prophylactic patch placement before subsequent pressure recordings, registered a low posterior mean (0.066), illustrating how decompression resets the perfusion deficit signal once the tension is relieved. Patient 42 reached a comparable risk band (posterior mean 0.522) yet avoided decompression, underlining the value of continued surveillance when pressure deficits persist without organ failure. Patients 30 and 40 similarly occupied elevated risk bands (posterior means 0.406 and 0.338, respectively) without developing ACS, illustrating that perfusion deficits may be necessary but not sufficient for ACS, given that additional factors such as organ reserve and timing likely modulate progression. The overlap between the highest-risk non-events and the lower-risk ACS case (patient 23) underscores the exploratory nature of this analysis and the need for larger cohorts to establish reliable decision thresholds. The wider intervals for ACS cases reflect the limited event count but remain above negligible risk levels.

## 4. Discussion

### 4.1. Principal Findings

The APP and MAP deficits captured the discriminative signal for ACS in this exploratory cohort. Notably, one ACS case (patient 23) developed organ dysfunction despite an immediate postoperative IAP of only 8 mmHg, which fell below the WSACS threshold, because the low MAP (38 mmHg) produced a substantial APP deficit. This case illustrates the clinical situation in which intermittent IAP-only surveillance would fail to identify impending ACS, a limitation that APP-based monitoring directly addresses. As a proof of concept, this case demonstrates that ACS risk assessment should incorporate the hemodynamic denominator (MAP) alongside IAP. The observation that both ACS cases exhibited markedly depressed perfusion pressures while the prophylactically decompressed patient returned to the low-risk band is physiologically plausible. However, with only two ACS events, these findings are hypothesis-generating and require prospective validation. We purposely avoided a larger feature set because permutation importance analyses suggested that perfusion deficits accounted for the majority of discriminative signal, and the simplicity of a two-predictor approach makes it easily reproducible in clinical practice.

### 4.2. Comparison with Prior Work

While ACS after CDH repair is rarely encountered, its occurrence has been reported in the literature [1,2], and our two cases contribute further evidence recognizing this potentially severe postoperative complication. In an analysis of the CDH patients included in the Canadian Association of Pediatric Surgery database, in five years, three cases of ACS were reported [1]. A systematic review analyzing 16 published papers reported six cases of ACS after CDH repair [2]. Considering the challenges of publishing isolated cases and small case series and the rarity of ACS following CDH repair, additional cases may remain underreported.

Our findings align with the existing literature suggesting that IAP alone does not adequately reflect the perfusion status, while APP deficits herald organ compromise [19,20]. Studies from Pediatric Intensive Care Units have reported ACS at IAP values between 12 and 18 mmHg at reduced MAP values, reinforcing the pressure-deficit approach analyzed in our study [17,37]. Horoz et al. demonstrated the association between APP and mortality in general pediatric intensive care population [20]. Our study extends these findings to CDH patients, in whom the disproportion between the volume of viscera herniated in the thoracic cavity and the underdeveloped abdominal cavity, creates a unique pathophysiology.

Thambusamy et al. observed that 83% of CDH neonates had elevated IAP on the first postoperative day, with IAH duration correlating with ventilation duration, reinforcing the clinical importance of early IAP monitoring [38].

International surveys regarding the awareness and management of intra-abdominal hypertension highlight the heterogeneity in acknowledgment of WSACS thresholds and routine monitoring; for example, some respondents do not use the appropriate priming volumes for IAP measurement, underscoring the need for the development of standardized diagnostic and therapeutic algorithms for children [39,40,41,42,43]. An electronic survey conducted by WSACS almost a decade after the publication of its latest guideline, highlighted that, from 1042 health care professionals from 102 countries who completed the survey, 41% were unfamiliar with the guideline [44].

Our results are also consistent with near-infrared spectroscopy (NIRS) and tissue reflectance spectrophotometry studies and experimental models suggesting that modest IAP elevations reduce splanchnic oxygen delivery, enhancing the physiological plausibility to APP-focused surveillance [18,45].

Whereas previous CDH studies focused on physiology or surgical technique without providing decision-ready risk estimates [1,2], our study provides calibrated probabilities and uncertainty intervals which can support decision-making and optimize outcomes.

### 4.3. Clinical Implications

**Figure 4:**
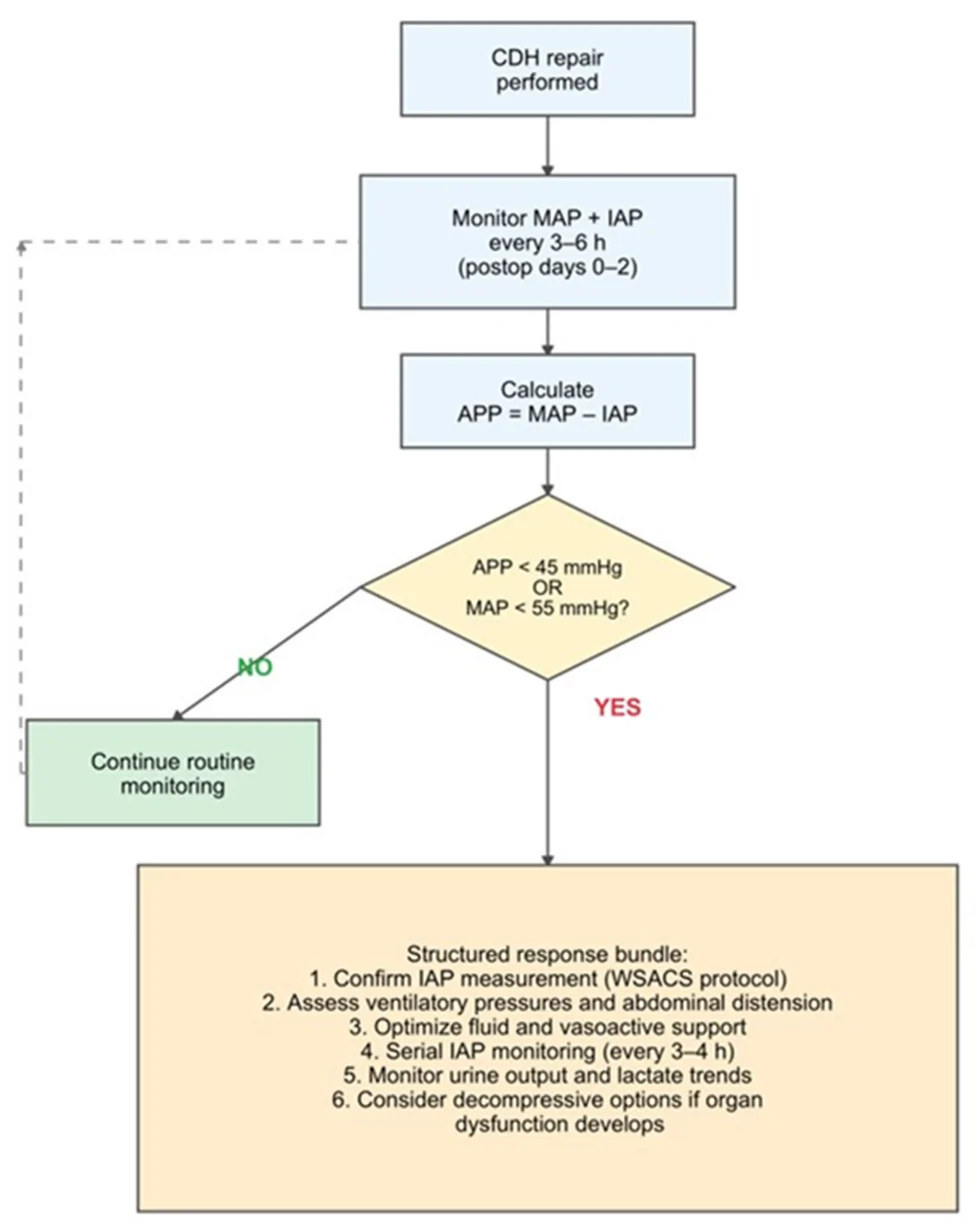
Proposed clinical surveillance pathway for postoperative perfusion deficit monitoring after CDH repair. APP, abdominal perfusion pressure; MAP, mean arterial pressure; IAP, intra-abdominal pressure; WSACS, World Society of the Abdominal Compartment Syndrome.

We propose a clinical surveillance pathway, illustrated in Figure 4, aimed at enabling early identification of patients at risk for developing ACS and to guide timely intervention.

While requiring prospective validation, the proposed clinical surveillance pathway translates our findings into a practical monitoring framework. Any non-zero deficit (MAP < 55 or APP < 45 mmHg) warrants heightened surveillance. Both ACS cases had deficits of approximately 17–19 mmHg, compared with mild deficits (median approximately 5–6 mmHg) in the non-ACS cohort. The model correctly identified both ACS cases at the top of the posterior ranking, whereas patient 37 (prophylactic dual-surface expanded polytetrafluoroethylene patch placed before pressures spiked) fell back into the low-risk band, and patient 42 remained a non-event despite several days of elevated IAP. The novelty of the proposed pathway lies in the formal threshold-based triggering of a structured response, rather than in the individual bundle components, which reflect established clinical practice.

A risk estimate above the threshold should trigger a structured bundle: confirm proper IAP measurement technique, relieve ventilatory or abdominal distension, optimize fluid and vasoactive support, observe urine output and lactate trends, and consider decompressive options. It should be noted that the pathway is based on physiologic thresholds, which represents a practical strength for bedside implementation. However, thresholds may need gestational-age adjustment for preterm infants. Embedding this assessment within routine postoperative monitoring requires no additional equipment, as MAP and IAP are already charted in CDH patients. These exploratory findings merit prospective evaluation before clinical implementation.

### 4.4. Strengths and Limitations

The strengths include prespecified physiologic estimators derived from established MAP targets for neonates and the IAP threshold defined by WSACS, near-complete data with one infant partially missing MAP/APP recordings, and a reproducible analytic pipeline.

The modest sample size (28 infants with two ACS events) produces wide confidence intervals and precludes model validation. The events-per-parameter ratio of 1 is well below the recommended minimum of 5. Neither ridge nor Firth penalization can produce reliable probability estimates at this sample size, and the ROC-AUC of 0.94 should be interpreted cautiously as it may reflect chance separation. The compact model could not reliably distinguish ACS patient 23 from three non-ACS patients with comparable deficits (patients 30, 40, 42), whose estimated probabilities were similar. The ROC-AUC is primarily driven by the correct ranking of the more severe ACS case (patient 46); with only two events, this metric should be interpreted as reflecting a consistent association rather than stable discrimination. We chose ridge logistic regression over Firth’s penalized likelihood [46,47] because the primary goal was discrimination rather than unbiased coefficient estimation; Firth’s method, while preventing separation, can bias estimated probabilities toward 0.5 in rare-event settings [48]. Future work with larger samples should compare both approaches.

Two patients who required extracorporeal membrane oxygenation (ECMO) support were included in the analysis. One patient underwent ECMO for 8 days, and the other underwent ECMO for 5 days. MAP values during ECMO are partly circuit-dependent, which may confound perfusion-deficit interpretation. Additionally, we did not include in the study echocardiographic data (such as fractional shortening or cardiac output indices) that could differentiate primary cardiogenic shock from compartment-driven hypoperfusion; patient 23, born with an Apgar score of 4 and a recorded MAP of 38 mmHg, may have had a component of primary cardiac dysfunction that independently depressed the MAP, exacerbating the perfusion deficit attributable to compartment pathology. In addition, the relationship between hemodynamic compromise and high intrathoracic pressure due to high ventilator settings that could lead to low MAP was not analyzed in this study. Given the physiological plausibility that high intrathoracic pressures reduce venous return and depress MAP, future studies should systematically record both PIP and PEEP alongside perfusion parameters. However, regardless of whether MAP depression is cardiogenic or compartment-driven, the resulting APP deficit identifies a state of compromised perfusion that may warrant heightened surveillance. Vasoactive medication type and dosing were not recorded in our study, representing an unmeasured confounder for MAP-based predictors.

Patient 37 underwent prophylactic decompression and was classified as non-ACS; if the intervention pre-empted ACS that would otherwise have developed, this represents outcome misclassification. Standardized CDH severity indices (observed/expected lung-to-head ratio, defect grade) were not uniformly available in the patients’ records and were not included in the study.

### 4.5. Future Directions

Future work should prioritize multicenter studies that record perfusion indicators (e.g., splanchnic NIRS, renal biomarkers) and prospectively evaluate the proposed surveillance pathway. At the observed 7.1% prevalence, assuming comparable ACS prevalence across centers, a multicenter cohort of approximately 140 CDH repairs would be needed to achieve 10 events, the minimum for stable 2-predictor model estimation. Larger datasets would enable external validation and development of time-varying postoperative risk trajectories.

Future multicenter studies are needed to define validated APP thresholds in the pediatric population, particularly in neonates given their distinct physiologic characteristics. This line of research could contribute to development of standardized monitoring protocols for children at risk of ACS.

## 5. Conclusions

Clinically significant deficits in perfusion pressure, specifically decreases in MAP and APP below physiologic safety thresholds, may serve as early warning signals for ACS after CDH repair in neonates. These exploratory findings require validation before clinical implementation. Furthermore, these observations support the development of a bedside surveillance pathway that should be considered in all neonates with potential viscero-abdominal disproportion that are susceptible to increased IAP (e.g., CDH, gastroschisis, omphalocele. This surveillance pathway warrants prospective multicenter evaluation.

## Figures and Tables

**Table 1:** Baseline characteristics and postoperative pressure parameters. Continuous variables are reported as median [IQR] and categorical variables as *n* (%). MAP deficit = shortfall below 55 mmHg; APP deficit = shortfall below 45 mmHg (zero, if target maintained).

Variable	Overall (*N* = 28)	Non-ACS (*n* = 26)	ACS (*n* = 2)
Gestational age, weeks	38.0 [36.8–39.0]	38.0 [37.2–39.0]	36, 39
Birth weight, g	3080.0 [2787.5–3342.5]	3100.0 [2812.5–3347.5]	2350, 3000
Male sex, *n* (%)	17 (60.7%)	17 (65.4%)	0 (0.0%)
Apgar score at 5 min	7.0 [5.8–8.0]	7.0 [6.0–8.0]	4, 5
Prenatal diagnosis, *n* (%)	25 (89.3%)	23 (88.5%)	2 (100.0%)
Age at surgery, hours	30.5 [22.8–48.0]	30.5 [20.2–48.0]	24, 48
Cesarean delivery, *n* (%)	22 (78.6%)	20 (76.9%)	2 (100.0%)
Open surgical approach, *n* (%)	24 (85.7%)	22 (84.6%)	2 (100.0%)
Patch repair, *n* (%)	9 (32.1%)	8 (30.8%)	1 (50.0%)
ECMO support, *n* (%)	2 (7.1%)	2 (7.7%)	0 (0.0%)
Initial IAP, mmHg	7.0 [6.0–9.0]	7.0 [6.0–9.0]	8, 11
IAP max (d0–2), mmHg	8.0 [6.8–9.6]	8.0 [6.2–9.4]	8, 11
MAP min (d0–2), mmHg	47.7 [43.5–50.3]	48.0 [44.0–50.7]	33, 38.3
APP min (d0–2), mmHg	40.0 [36.5–44.0]	40.3 [38.3–44.0]	22, 33.3
MAP deficit (mmHg) †	7.2 [4.1–11.4]	6.3 [3.6–10.9]	16.7, 22
APP deficit (mmHg) †	4.8 [0.8–8.4]	4.7 [0.5–6.6]	11.7, 23

ACS, abdominal compartment syndrome; ECMO, extracorporeal membrane oxygenation; IAP, intra-abdominal pressure; MAP, mean arterial pressure; APP, abdominal perfusion pressure; † MAP deficit was calculated as the difference between the target 55mmHg MAP and the minimum daily MAP; APP deficit was calculated as the difference between the target 45 mmHg APP and the minimum daily APP.

**Table 2:** Penalized logistic model coefficients (full-sample fit) on raw (unstandardized) scales. APP deficit = shortfall of minimum APP below 45 mmHg; MAP deficit = shortfall of minimum MAP below 55 mmHg. Odds ratios are derived from ridge-penalized coefficients and are shrunk toward 1.0 to prevent overfitting; they represent conservative lower-bound estimates rather than traditional unpenalized maximum-likelihood effect sizes.

Term	Log-Odds Coefficient	Odds Ratio (per mmHg)
Intercept	−1.192	-
APP deficit (per mmHg)	0.059	1.06
MAP deficit (per mmHg)	0.073	1.08

MAP: mean arterial pressure; APP: abdominal perfusion pressure.

## Data Availability

All tables, figures, and summary statistics in this manuscript are generated from immutable CSV/JSON inputs. All anonymized data alongside the processing scripts provides a complete reproducible workflow, which we can share upon request.

## Supplementary Materials

The following supporting information can be downloaded at https://www.mdpi.com/article/10.3390/life16091496/s1, Supplementary Table S1: Bayesian GLM posterior means and 95% credible intervals on raw scales; Supplementary Table S2: Performance summary comparing penalized logistic (compact) and Bayesian models (LOOCV); Supplementary Table S3: Operating points at high-specificity thresholds (LOOCV); Supplementary Table S4: Calibration summary with bootstrap 95% Cis; Supplementary Figure S1: Decision-curve analysis showing net benefit versus alert threshold; Supplementary Table S5: Decision tree rules and training performance; Supplementary Table S5b: Decision tree overall training performance; Supplementary Figure S2: VISTLA variable-importance network showing relationships between pressure-derived features and ACS outcome; Supplementary Figure S3: Decision tree diagram for ACS classification; Supplementary Table S6: SIMEX measurement-noise stress-test results showing model discrimination under increasing noise levels; Supplementary Table S7: Synergy threshold sweep exploring joint IAP/MAP burden across different threshold combinations; Supplementary Table S8: Feature summaries for d0–2 pressure minima and perfusion deficits by ACS group; Supplementary Figure S4. Bedside monitoring system displaying patient’s 47 signs including intravesical pressure labeled as UVP.

## Abbreviations

The following abbreviations are used in this manuscript:

ACS	Abdominal compartment syndrome
APP	Abdominal perfusion pressure
CDH	Congenital diaphragmatic hernia
ECMO	Extracorporeal membrane oxygenation
IAP	Intra-abdominal pressure
LOOCV	Leave-one-out cross-validation
MAP	Mean arterial pressure
NIRS	Near-infrared spectroscopy
POD	Postoperative day
ROC-AUC	Receiver operating characteristic area under the curve
STROBE	Strengthening the Reporting of Observational Studies in Epidemiology
WSACS	World Society of the Abdominal Compartment Syndrome
