# Supplementary material for: Perfusion Pressure Deficits as Early Indicators of Abdominal Compartment Syndrome After Neonatal Congenital Diaphragmatic Hernia Repair: A Retrospective Cohort Study
Source: Life (Basel). 2026 Sep 7;16(9):1496. doi: 10.3390/life16091496 (PMC13608666; doi:10.3390/life16091496)
Supplement: Supplementary file 1 [file life-16-01496-s001.zip › life-4480329-supplementary.pdf]

Perfusion Pressure Deficits as Early Indicators of Abdominal Compartment Syndrome After Neonatal Congenital Diaphragmatic Hernia Repair: A Retrospective Cohort Study

Supplementary material

A Bayesian generalized linear model (GLM) was fit with weakly informative directional priors (normal(0, 2) truncated at zero) on the deficit predictors plus supportive physiologic descriptors (synergy integral, diuresis burden, mean airway pressure, immediate post repair IAP). The model generated 10,000 posterior draws per patient to characterize uncertainty, and convergence was confirmed via standard convergence diagnostics (potential scale reduction factors <1.01 and effective sample sizes >1,000 for all parameters).

The Bayesian GLM incorporated supportive physiologic descriptors with shrinkage priors; their posterior means and approximate 95% credible intervals remain centered near zero relative to the deficit effects, underscoring the dominance of perfusion deficits (Table 1).

Supplementary Table S1. Bayesian GLM posterior means and 95% credible intervals on raw scales.

Term	Log-odds mean (per unit)	95% CrI (log-odds)	Odds ratio (95% CrI)
Intercept	-3.189	-4.540 to -1.838	---
APP deficit (per mmHg)	0.086	-0.024 to 0.197	1.09 (0.98--1.22)
MAP deficit (per mmHg)	0.103	-0.012 to 0.217	1.11 (0.99--1.24)
Synergy integral (per mmHg*h)	0.022	-0.013 to 0.057	1.02 (0.99--1.06)
Diuresis AUC <2 mL/kg/h (per h)	0.500	-0.284 to 1.284	1.65 (0.75--3.61)
Mean airway pressure (per cmH2O)	0.049	-0.100 to 0.199	1.05 (0.90--1.22)
Immediate postrepair IAP (per mmHg)	0.034	-0.276 to 0.344	1.03 (0.76--1.41)

Supplementary Table S2. Performance summary comparing penalized logistic (compact) and Bayesian models (LOOCV).

Model	ROC-AUC (95% CI)	PR-AUC (95% CI)	Brier score
Compact (LOOCV)	0.942 (0.808--1.000)	0.700 (0.000--1.000)	0.194
Bayesian (LOOCV)	0.885 (0.741--1.000)	0.333 (0.000--1.000)	0.088

Supplementary Table S3. Operating points at high-specificity thresholds (LOOCV).

Model	Threshold (pt)	TP	FP	TN	FN	Sensitivity (95% CI)	Specificity (95% CI)	PPV (95% CI)	NPV (95% CI)	Alert rate
Compact	0.75	1	2	24	1	0.50 (0.00--1.00)	0.92 (0.80--1.00)	0.33 (0.00--1.00)	0.96 (0.88--1.00)	0.11
Compact	0.79	1	2	24	1	0.50 (0.00--1.00)	0.92 (0.80--1.00)	0.33 (0.00--1.00)	0.96 (0.88--1.00)	0.11
Compact	0.85	1	0	26	1	0.50 (0.00--1.00)	1.00 (1.00--1.00)	1.00 (1.00--1.00)	0.96 (0.88--1.00)	0.04
Bayesian	0.75	0	1	25	2	0.00 (0.00--0.00)	0.96 (0.88--1.00)	0.00 (0.00--0.00)	0.93 (0.81--1.00)	0.04
Bayesian	0.79	0	1	25	2	0.00 (0.00--0.00)	0.96 (0.88--1.00)	0.00 (0.00--0.00)	0.93 (0.81--1.00)	0.04
Bayesian	0.85	0	1	25	2	0.00 (0.00--0.00)	0.96 (0.88--1.00)	0.00 (0.00--0.00)	0.93 (0.81--1.00)	0.04

At an alert rate of 0.036 (threshold 0.85) the penalized model flagged one of two ACS cases with no false positives (specificity 1.00); relaxing the threshold to 0.754 increased the alert rate to 0.11 and introduced two false positives while maintaining 0.50 sensitivity.

Supplementary Table S4. Calibration summary with bootstrap 95% CIs. ECE, expected calibration error.

Model	Intercept (95% CI)	Slope (95% CI)	ECE (8 bins)
Compact (pre)	-2.67 (-25.54–-1.62)	2.16 (0.00–116.16)	0.372
Compact (post)	Not estimable	1.60 (0.00–3.87)	0.000
Bayesian	-0.53 (-24.40–0.88)	1.17 (0.00–137.11)	0.057

LOOCV calibration yielded intercept/slope not estimable/1.60 for the penalized model and -0.53/1.17 for the Bayesian model, with ECE of 0 and 0.057 respectively. The slightly sub-unity slopes highlight residual over-shrinkage, yet both curves hugged the 45-degree line within sampling error, especially after probability recalibration.

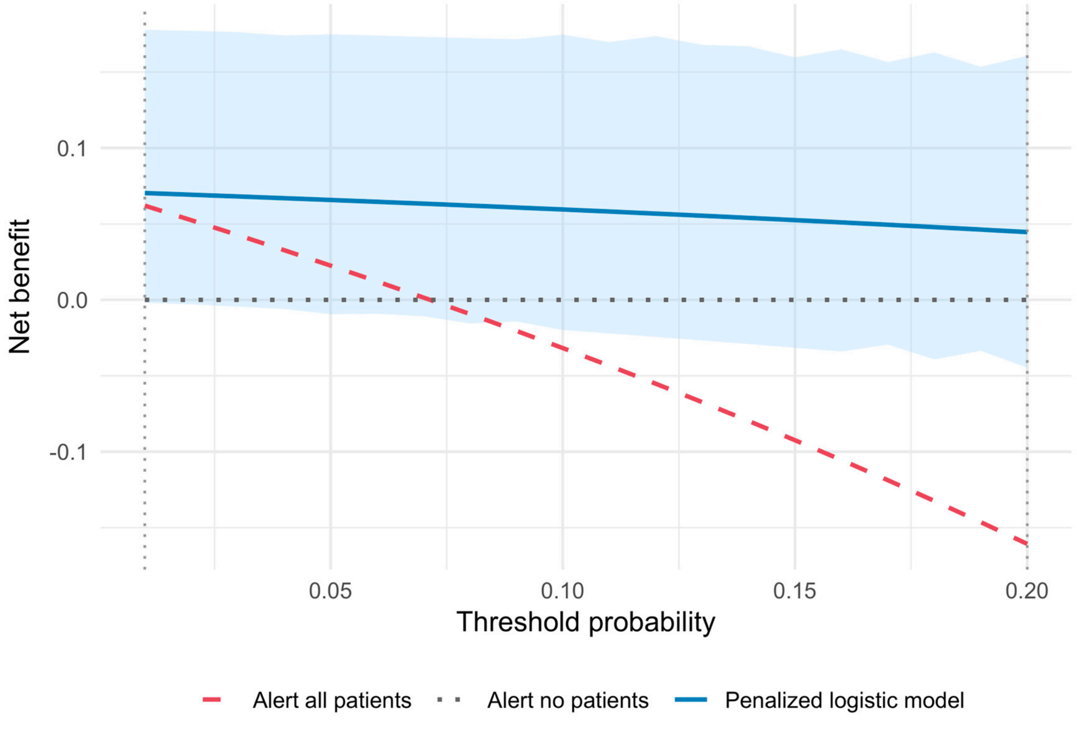

Supplementary Figure S1. Decision-curve analysis showing net benefit versus alert threshold. The penalized logistic model (blue line, ribbon = bootstrap 95% CI) is compared against strategies of alerting every patient (red dashed) or never alerting (grey dotted). Vertical dotted lines mark the positive net benefit range.

Decision-curve analysis interprets model output as clinical payoff: for each alert threshold on the x-axis it computes net benefit, the proportion of true positives minus a penalty for false positives weighted by the chosen threshold. For thresholds between 0.01 and 0.2 the model maintained positive net benefit relative to treat-none and stayed above treat-all in the 0.01–0.20 region that matters for rare events [1,2]. The wide confidence intervals reflect the limited event count and indicate that the net benefit estimates are not statistically distinguishable from the treat-none strategy at most individual thresholds, though the model consistently trends above treat-none across the entire clinically relevant range.

Supplementary Table S5. Decision tree rules and training performance.

leaf	N	Coverage	Positives	Positive rate	Positive rate 95% CI	Predicted class
L1: map_auc<=14.83 & synergy<=1.67 -> NoACS	20	71.4%	0	0.0	0.00--0.17	NoACS
L2: map_auc<=14.83 & synergy>1.67 -> NoACS	3	10.7%	0	0.0	0.00--0.71	NoACS
L3: map_auc>14.83 & MAP deficit<=19.5 -> ACS	2	7.1%	1	0.5	0.01--0.99	ACS
L4: map_auc>14.83 & MAP deficit>19.5 -> ACS	2	7.1%	1	0.5	0.01--0.99	ACS
Unassigned	1	3.6%	0	0.0	0.00--0.97	NoACS

Supplementary Table S5b. Decision tree overall training performance.

Metric	Estimate	95% CI
Sensitivity	1.000	0.16--1.00
Specificity	0.923	0.75--0.99

A shallow decision tree was constructed using pressure-derived features, highlighting the dominant pressure-first relationships. Training sensitivity was 1 (0.16–1.00) and specificity 0.92 (0.75–0.99).

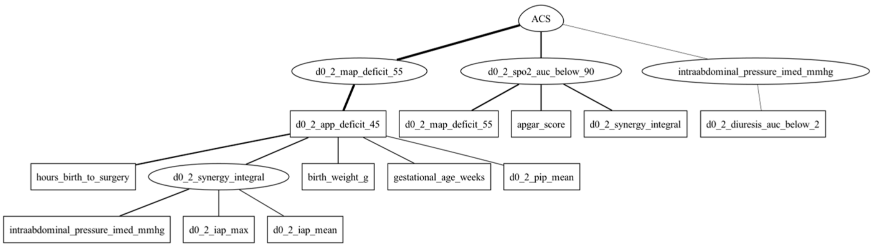

Supplementary Figure S2. VISTLA variable-importance network showing relationships between pressure-derived features and ACS outcome.

The variable-importance network maps the densest links between abdominal compartment syndrome and the APP/MAP deficits, confirming that perfusion-focused features dominate the discriminative signal.

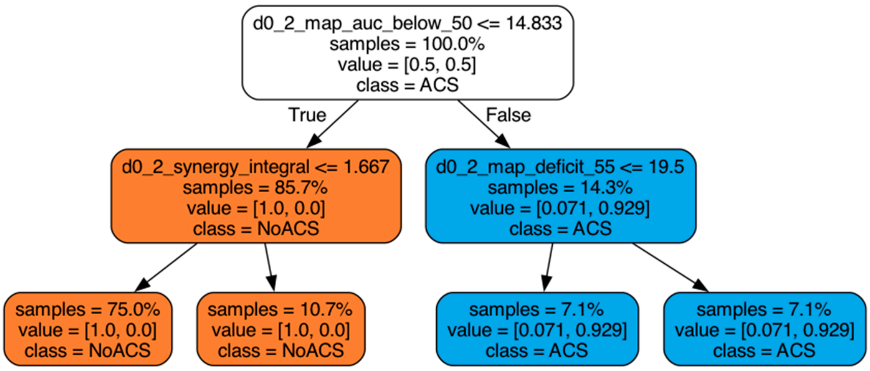

Supplementary Figure S3. Decision tree diagram for ACS classification.

Supplementary Table S6. SIMEX measurement-noise stress-test results showing model discrimination under increasing noise levels.

scenario	Category	iap_ shift	map_ shift	noise_ sigma	repli cates	mean_p ositive_ risk	mean_p ositive_ risk_sd	mean_ negati ve_ris k	mean_ negati ve_ris k_sd
baseline	Baseline	0.0	0.0	0.0	1	0.76123 10	0.000000 00	0.41505 58	0.00000 00
iap_+0.5	Iap	0.5	0.0	0.0	1	0.76629 88	0.000000 00	0.42045 16	0.00000 00
iap_-0.5	Iap	-0.5	0.0	0.0	1	0.75609 24	0.000000 00	0.40999 12	0.00000 00
map_- 0.5	Map	0.0	-0.5	0.0	1	0.77244 71	0.000000 00	0.42712 06	0.00000 00
both_+0. 5	Both	0.5	-0.5	0.0	1	0.77735 50	0.000000 00	0.43254 36	0.00000 00
both_- 0.5	Both	-0.5	0.5	0.0	1	0.74436 72	0.000000 00	0.39849 69	0.00000 00
iap_+1.0	Iap	1.0	0.0	0.0	1	0.77129 52	0.000000 00	0.42586 07	0.00000 00
iap_-1.0	Iap	-1.0	0.0	0.0	1	0.75088 35	0.000000 00	0.40509 50	0.00000 00
map_- 1.0	Map	0.0	-1.0	0.0	1	0.78330 50	0.000000 00	0.43924 05	0.00000 00
both_+1. 0	Both	1.0	-1.0	0.0	1	0.79272 46	0.000000 00	0.45012 77	0.00000 00
both_- 1.0	Both	-1.0	1.0	0.0	1	0.72678 50	0.000000 00	0.38346 62	0.00000 00
iap_+2.0	Iap	2.0	0.0	0.0	1	0.78107 23	0.000000 00	0.43671 15	0.00000 00
iap_-2.0	Iap	-2.0	0.0	0.0	1	0.74025 75	0.000000 00	0.39659 03	0.00000 00

map_-2.0	Map	0.0	-2.0	0.0	1	0.8039335	0.00000000	0.4637310	0.0000000
both_+2.0	Both	2.0	-2.0	0.0	1	0.8211756	0.00000000	0.4855015	0.0000000
both_-2.0	Both	-2.0	2.0	0.0	1	0.6895862	0.00000000	0.3541464	0.0000000
noise_iap_sigma0.5	noise_iap	0.0	0.0	0.5	20	0.7607800	0.09261481	0.4152793	0.1594707
noise_map_sigma0.5	noise_map	0.0	0.0	0.5	20	0.7610363	0.09374955	0.4155103	0.1599915
noise_both_sigma0.5	noise_both	0.0	0.0	0.5	20	0.7604970	0.09438346	0.4157537	0.1602300
noise_iap_sigma1.0	noise_iap	0.0	0.0	1.0	20	0.7615669	0.09288978	0.4156047	0.1590852
noise_map_sigma1.0	noise_map	0.0	0.0	1.0	20	0.7545439	0.10142728	0.4161050	0.1598475
noise_both_sigma1.0	noise_both	0.0	0.0	1.0	20	0.7594233	0.09472495	0.4170169	0.1604852
noise_iap_sigma2.0	noise_iap	0.0	0.0	2.0	20	0.7628786	0.09966168	0.4180438	0.1597067
noise_map_sigma2.0	noise_map	0.0	0.0	2.0	20	0.7528068	0.09476928	0.4197160	0.1633933
noise_both_sigma2.0	noise_both	0.0	0.0	2.0	20	0.7531053	0.10910494	0.4252495	0.1605347

Deterministic and stochastic perturbations of MAP/IAP (SIMEX-style) confirmed that modest measurement noise does not erode discrimination.

Supplementary Table S7. Synergy threshold sweep exploring joint IAP/MAP burden across different threshold combinations.

iap_thr	map_thr	roc_auc	pr_auc	mean_synergy_pos	mean_synergy_neg	cliffs_delta_approx	mwu_p_value
8	45	0.7211538	0.53571429	18.00000	1.6730769	0.44230769	0.10696797
8	50	0.6730769	0.28571429	25.50000	4.1282051	0.34615385	0.31882524
8	55	0.6634615	0.28571429	38.66667	9.8141026	0.32692308	0.37087179
8	60	0.6634615	0.28571429	53.66667	16.3589744	0.32692308	0.37087179
9	45	0.7211538	0.53571429	12.00000	1.1089744	0.44230769	0.10696797
9	50	0.6923077	0.28571429	17.00000	2.6666667	0.38461538	0.20450109
9	55	0.6826923	0.28571429	24.33333	6.3269231	0.36538462	0.26340233
9	60	0.6826923	0.28571429	31.83333	10.4615385	0.36538462	0.26340233
10	45	0.7211538	0.28571429	6.00000	0.5769231	0.44230769	0.02803017
10	50	0.7115385	0.28571429	8.50000	1.4358974	0.42307692	0.08116137
10	55	0.6923077	0.28571429	11.00000	3.5512821	0.38461538	0.20450109
10	60	0.6923077	0.28571429	13.50000	5.8589744	0.38461538	0.20450109

11	45	0.4807692	0.07142857	0.00000	0.3846154	-0.03846154	0.88970694
11	50	0.4807692	0.07142857	0.00000	0.8589744	-0.03846154	0.88970694
11	55	0.4615385	0.07142857	0.00000	1.9487179	-0.07692308	0.76450338

Synergy threshold sweeps examined joint IAP/MAP burden, confirming that perfusion-focused thresholds dominate performance across a range of parameter combinations.

Supplementary Table S8. Feature summaries for d0–2 pressure minima and perfusion deficits by ACS group (median [IQR]).

**Group**	**MAPmin (mmHg)**	**APPmin (mmHg)**	**MAP deficit (mmHg)**	**APP deficit (mmHg)**
Overall	47.7 [43.5–50.3]	40.0 [36.5–44.0]	7.2 [4.1–11.4]	4.8 [0.8–8.4]
ACS	35.7 [34.3–37.0]	27.7 [24.8–30.5]	19.3 [18.0–20.7]	17.3 [14.5–20.2]
No ACS	48.0 [44.0–50.7]	40.3 [38.3–44.0]	6.3 [3.6–10.9]	4.7 [0.5–6.6]

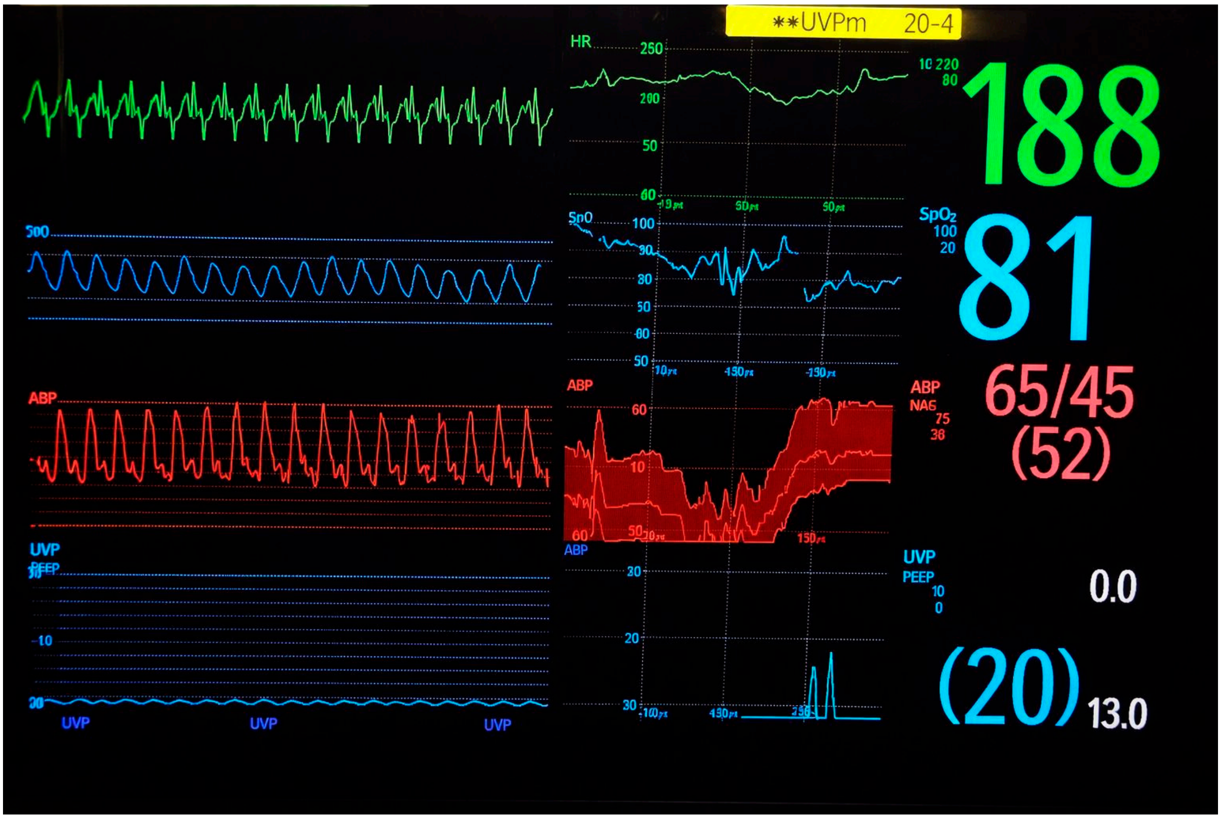

Supplementary Figure S4. Bedside monitoring system displaying patient's 47 signs including intravesical pressure labeled as UVP
